# Various Approaches to Studying the Phase Transition in an Octamethylcyclotetrasiloxane Crystal: From X-ray Structural Analysis to Metadynamics

**DOI:** 10.3390/ijms23169073

**Published:** 2022-08-13

**Authors:** Alexander D. Volodin, Alexander F. Smol’yakov, Alexander A. Korlyukov

**Affiliations:** 1 A. N. Nesmeyanov Institute of Organoelement Compounds, Russian Academy of Science, Vavilova Street 28, 119991 Moscow, Russia; 2 Highest Chemical College of RAS, D. Mendeleev University of Chemical Technology of Russia, Miusskaya Square 9, 125047 Moscow, Russia; 3 Basic Department of Chemistry of Innovative Materials and Technologies, Plekhanov Russian University of Economics, Stremyannyy Pereulok 36, 117997 Moscow, Russia

**Keywords:** in situ cryo-crystallization, X-ray structure determination, periodic DFT calculations, quantum chemical calculations, Born–Oppenheimer molecular dynamics, metadynamics, phase transition path, free energy of phase transition

## Abstract

The structure, thermodynamic parameters, and the character of thermal motion in octamethylcyclotetrasiloxane (D4) were investigated using the combination of experimental (single-crystal X-ray diffraction, thermochemistry) and theoretical (density functional theory calculations, ab initio molecular dynamics and metadynamics) methods. Single crystals of D4 were grown in a glass capillary in situ and the structures of high- (238–270 K) and low-temperature (100–230 K) phases were studied in detail. In the temperature interval 230–238 K, a phase transition with rather low enthalpy (−1.04(7) kcal/mol) was detected. It was found that phase transition is accompanied by change of conformation of cyclosiloxane moiety from boat-saddle (cradle) to chair. According to PBE0/6-311G(d,p) calculation of isolated D4, such conformation changes are characterized by a low barrier (0.07 kcal/mol). The character of molecular thermal motion and the path of phase transition were established with combination of periodic DFT calculations, including molecular dynamics and metadynamics. The effect of crystal field led to an increase in the calculated phase transition barrier (4.27 kcal/mol from low- to high-temperature phase and 3.20 kcal/mol in opposite direction).

## 1. Introduction

Cyclic oligosiloxanes are of great experimental and theoretical interest due to their wide practical use [1,2,3,4,5,6]. Siloxanes are used in various fields of medical, catalytic, and synthetic chemistry. The research results are used in various fields of industry and medicine. Siloxane monomers usually are viscous colorless liquids. The physical macroscopic properties (viscosity, thermal expansion, heat capacity, temperatures of phase transitions, etc.) of these compounds are well studied, whereas their microscopic ones (liquid microstructure and crystal structures, Debye–Waller factors, etc.) are studied to a lesser extent. Detailed study of microscopic properties is difficult and highly desirable to be carried out together with the use of atomistic modeling in combination with precise experimental measurements by diffraction and thermochemical techniques.

**Scheme 1:**
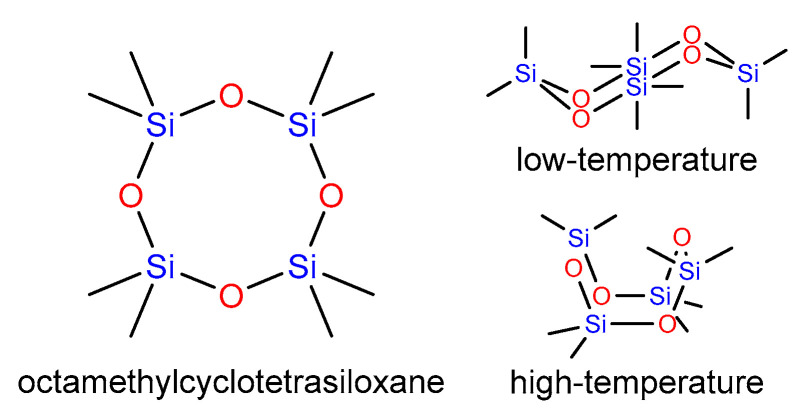
Octamethylcyclotetrasiloxane and it’s conformations in different crystal phases.

The octamethylcyclotetrasiloxane (D4, [Me_2_SiO]_4_, Scheme 1) attracted our interest due to its molecular flexibility and great importance in the industry of various commercial products, including polymeric materials and lubricants. Hence, the structure and properties of D4 have been discussed in several publications. Indeed, in 1953, the dependence of the dielectric constant on temperature was studied by J. Hoffman [7], who found a phase transition at −16.3 °C. Two years later, in 1955, H. Steinfink et al. carried out a series of X-ray diffraction experiments and established the crystalline structure of the low-temperature phase at −50 °C [8]. Their attempt to determine the structure of the high-temperature phase of D4 was unsuccessful; they were only able to determine the spacegroup (*I*4_1_/*n*) and unit cell parameters. The authors suggested that molecules in a crystal with *I*4_1_/*n* symmetry should be located on the $\bar{4}$ axes and should probably be disordered. Later, Shklover et al. showed that the structure of the high-temperature phase can be ordered, and the boat-saddle conformation (S_4_ symmetry) is the most possible for an octa-membered cyclosiloxane ring in contrast to the chair conformation (C_2h_ symmetry) in the low-temperature phase [9].

One of the most reliable approaches to obtain the molecular structure of a low-melting compound consists of growing a crystal in situ on an X-ray diffractometer and performing subsequent diffraction experiments [10,11,12]. Inspired by the works of R. Boese, W. Lipscomb, and M. Antipin, we decided to design our in-house heating device suitable for in situ crystallization using nichrome wire as a heater [13,14,15,16,17,18,19].

The reliable approaches to study the process pathway are known [20,21,22,23,24,25,26,27,28]. To determine the crystal structure of the high-temperature phase and gain an insight into the mechanism of phase transition, we carried out the X-ray and differential scanning calorimetry (DSC) study of D4. We performed X-ray experimental series of D4 on a monocrystal diffractometer with in situ crystallized samples. The combination of X-ray diffraction analysis and computational methods allow us to establish energy parameters and the pathway of phase transition.

## 2. Results and Discussion

### 2.1. The Crystal Structure of the Low- and High-Temperature Phases of Octamethylcyclotetrasiloxane

At room temperature, octamethylcyclotetrasiloxane is a transparent liquid with fairly high viscosity. Despite the preliminary drying of the D4, the first attempts of crystallization upon gradual cooling of a capillary were unsuccessful. Indeed, after using the zone melting procedure, D4 formed an amorphous phase or a polycrystalline sample consisting of numerous domains. This fact can be explained by overcooling of liquid D4 and ensuing rapid crystallization or solidification process far from equilibrium (up to 40 °C). To decrease the effect of overcooling and then bring crystallization closer to equilibrium, an external nucleation initiator (a tiny NaCl crystal) was added. The addition of an external nucleating agent resulted in accelerated crystallization and a significant decrease in the number of crystals. As a result, a single crystal suitable for X-ray diffraction was grown at 270 K. The crystal structure of D4 was studied at 270, 250, 240, 237, 230, 227, 224, 221, 210, 200, 150, and 100 K.

**Figure 1:**
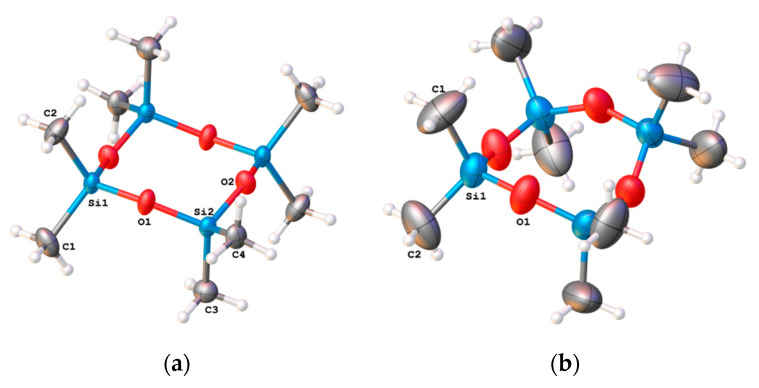
Conformations in low- (**a**) and high-temperature (**b**) phases. Ellipsoids are set at 50% probability.

The spacegroup and unit cell parameters in the high-temperature phase we studied at 270 K completely coincided with Steinfink’s observations (s.g. *I*4_1_/*a*, *a* = *b* = 16.250(7) Å, *c* = 6.943(4) Å). However, in contrast to Steinfink’s assumption, we found the molecular structure of D4 in the high-temperature phase to be ordered. The asymmetrical part of the unit cell contains a quarter of the D4 molecule (atoms Si1, O1, C1, and C2, Figure 1) since the molecule occupies a fourth-order position (on the $\bar{4}$ axis).

The highest value of the equivalent atom displacement parameter (U_eq_) for carbon atoms at 270 K is 0.13 Å^2^. At the same time, the positions of carbon atoms could not be separated without applying strong restraints (EADP and DFIX) due to the absence of reflections at high 2θ values (>50°). The U_eq_ values of oxygen and silicon atoms do not exceed 0.1 Å^2^. The shapes of atomic displacement parameter (ADP) ellipsoids are far from spherical; they are considerably stretched in a perpendicular (Si and O atoms) or tangential (C atoms) direction to the mean plane of the siloxane ring. Such behavior of ADP ellipsoids is indicative of the intensive molecular vibrations related to the folding of the siloxane cycle. As a consequence of thermal motion, Si1-O1 and Si1-O1[5/4−y,1/4 + x, 5/4−z] bonds (1.611(2) and 1.623(2) Å at 240 K) are somewhat shorter than those published in 1955 by Steinfink et al. for the low-temperature phase (1.64–1.66 Å). The length of Si1-C1 and Si1-C2 bonds (1.835(3) and 1.822(4) Å) also turned out to be shorter than those published earlier [8] by 0.07–0.12 Å.

**Figure 2:**
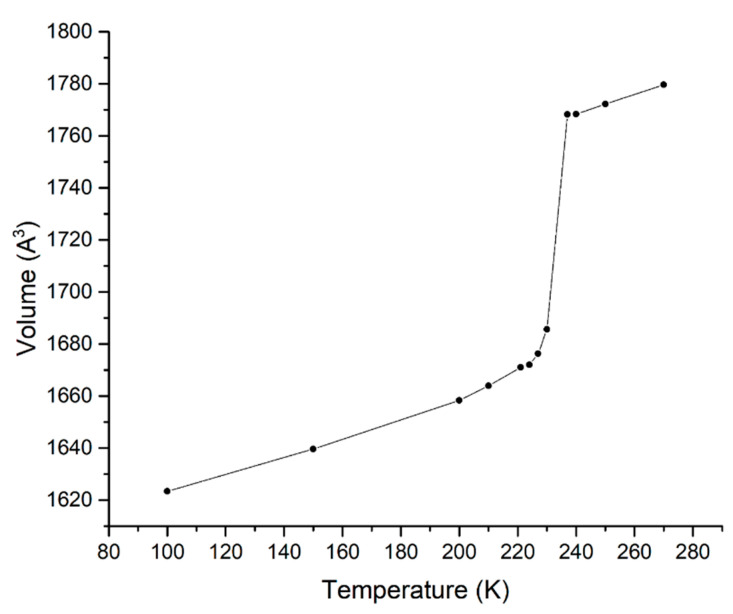
The dependence of unit cell volume on temperature.

The cell volume linearly decreased during cooling up to 238 K (Figure 2). The phase transition was observed at the temperature range between 230 and 238 K. Indeed, the picture of diffraction changed drastically at temperatures near 237–238 K; the number of reflections was considerably increased while those shapes were transformed from almost spherical to elliptical. The diffraction patterns came to resemble a pattern of a polycrystalline sample. Such a change led to an increase of R-values and the appearance of reflections, which should be absent for the *I*4_1_/*a* spacegroup. At 230 K and below, we found the diffraction pattern to be definitely indexing in the *P*4_2_/*n* spacegroup. To overcome the problems related to diffraction quality below 230 K, we studied several samples and various rates of cooling. 

The most accurate structural data were obtained for a crystal grown at a temperature of about 270 K and cooled at a rate of 60 °C/h. Surprisingly, at a cooling rate slower than 60 °C/h during the phase transition, the crystal is partially or completely destroyed. In this case, the diffraction pattern changes and becomes similar to the diffraction of a powder or even an amorphous body.

The X-ray study allowed us to establish the temperature of the phase transition with sufficient accuracy. According to data obtained, the phase transition occurs at 236–238 K on cooling and 240–242 K on heating of crystal samples of D4. Thus, the temperature of the phase transition in the capillary appears to be lower than the previously established temperature (257 K).

**Figure 3:**
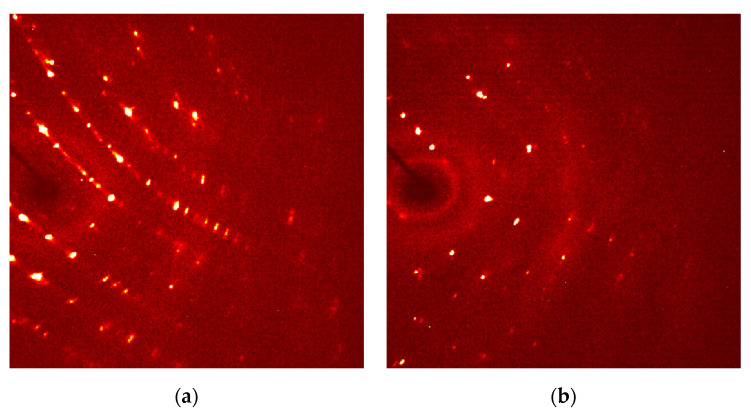
Diffraction patterns for low-temperature (**a**) and high-temperature (**b**) phases. Reflections corresponding to I-centering are absent in the high-temperature phase. Upon transition to the low-temperature phase, the reflections broadened.

The crystal structure of the low-temperature phase was the same as that described by Steinfink; however, we obtained more accurate atomic coordinates and displacement parameters. Cooling the sample to 100 K caused a considerable decrease in thermal motion and subsequent lowering of ADPs. At the same time, the differences between our study and the data published by Steinfink et al. in most cases were insignificant. The only exception was the lengths of Si-C bonds, which differ more strongly (1.850(3)–1.855(3) Å in our study vs 1.90–1.95 Å in [8]). As a result of phase transition, I-centering disappeared, and the fourfold position of the D4 molecule transformed into the center of inversion (Figure 3). Consequently, the change of spacegroup from *I*4_1_/*a* to *P*4_2_/*n* can lead to the formation of a disordered structure or a conformation transition. Indeed, the conformation of the eight-membered siloxane cycle in the high-temperature phase of D4 can be described as a boat-saddle, while that in the low-temperature phase presented as a chair conformation.

**Figure 4:**
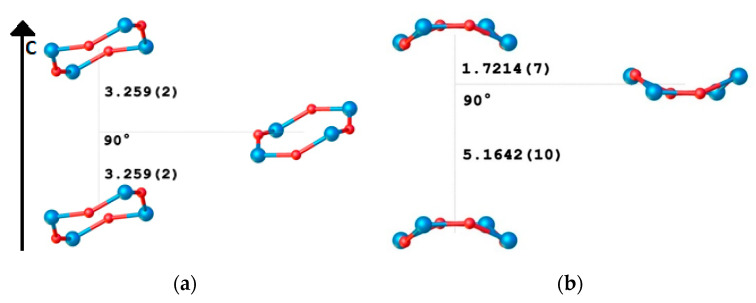
Mutual position of adjacent molecules in the low-temperature (**a**) and high-temperature (**b**) phases. Image plane equal to the ac plane of the unit cell. Hydrogen and carbon atoms are omitted for clarity. Color code: silicon—blue, oxygen—red.

In the low-temperature phase of D4, the z-coordinates (along c-axis) of centers of two adjacent molecules alternate by half the cell parameter c (3.259 Å). Concurrently, the z-coordinates of centers of two adjacent molecules differ by one-quarter of the cell parameter c (1.721 Å) in the high-temperature phase. Thus, the molecules should move at least 1.53 Å during the phase transition (Figure 4). According to Landau’s theory of phase transition types [29], D4 has a shift-type phase transition.

### 2.2. Differential Scanning Calorimetry Studies

**Figure 5:**
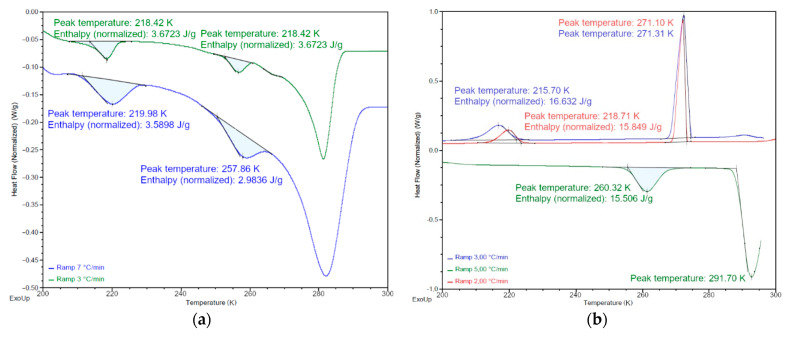
Differential scanning calorimetry data of impure (**a**) (98%) and pure (**b**) (>99.5%) D4 samples.

Two series of differential scanning calorimetry (DSC) experiments were performed for compound D4. The first experiments were carried out with an impure sample (98% purity, Figure 5a). We observed three peaks of differential heat on DSC: at 218–220, 256–258, and 280 K (melting point). The other one was performed with a pure sample (>99.5% purity, Figure 5b). In the second experiment, we only observed two phases and a huge hysteresis loop (>40 °C). At the same time, in an X-ray diffraction experiment, only two phases could be found. Thus, the phase that occurred at a temperature interval from 218 to 258 K is no other than a strange hysteresis loop, which should be a result of a slow asynchronous phase transition. Such a huge difference between DSC data shows how many problems could be caused by sample impurity.

The average enthalpy of solid–solid phase transition, calculated from the results of the DSC study, was 1.04(7) kcal/mol, while the calculated difference in total energy between the two solid phases was 3.14 kcal/mol (at 0 K, from VASP optimization).

### 2.3. The Comparison of Experimental and Calculated ADPs

Comparison of calculated and experimental atom displacement parameters is one of the easiest ways to find anomalies in molecular behavior. Besides, the ADP calculation method can be used to optimize strongly disordered structures and estimate the ability of molecules to move in a crystal. ADPs calculated with molecular dynamics were calculated and compared with experimental data. The average values of experimental and calculated $U_{eq}$ by type of atom (Equation (3)) are listed in Table 1.

The ADPs obtained from molecular dynamics (MD) calculations $\langle U_{eq}^{calc}\rangle$ at 100–200 K completely coincide with the experimentally measured ADPs $\langle U_{eq}^{exp}\rangle$. The $\langle U_{eq}^{calc}\rangle$ obtained at higher temperature values stand out from the general dependence. This happens because conformations significantly change and whole molecules move during MD calculation. Calculated molecule trajectories at 210 K and higher contain an intensive oscillation with a period near 4 ps (picoseconds), as described earlier. It causes large $\langle U_{eq}^{calc}\rangle$ values and outliers of this data from main dependence. In this case, the equivalent ADP values cannot be calculated correctly for all temperatures where the oscillation is present (210–250 K).

Another problem that happened during MD calculation was that the crystal system always suffers phase transition during MD calculations of the high-temperature phase (240, 250, and 270 K). The conformations and positions of molecules at the end of their MD trajectories almost coincide with those found in the calculated local minimum of the potential energy surface (PES). Thus, the comparison of ADPs at these temperatures with experimental ones is pointless, because the crystal structures correspond to different phases. The ADP values are high due to intense molecular motion. Molecules change conformations all the time during the simulation and oscillate distinctly along the c-axis.

### 2.4. Phonon Spectra and Physical Properties

**Figure 6:**
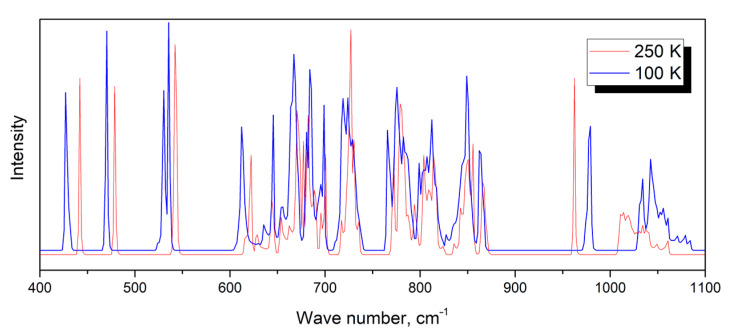
Comparison of the low- (blue) and high-temperature (red) phonon spectra at 100 and 250 K.

**Figure 7:**
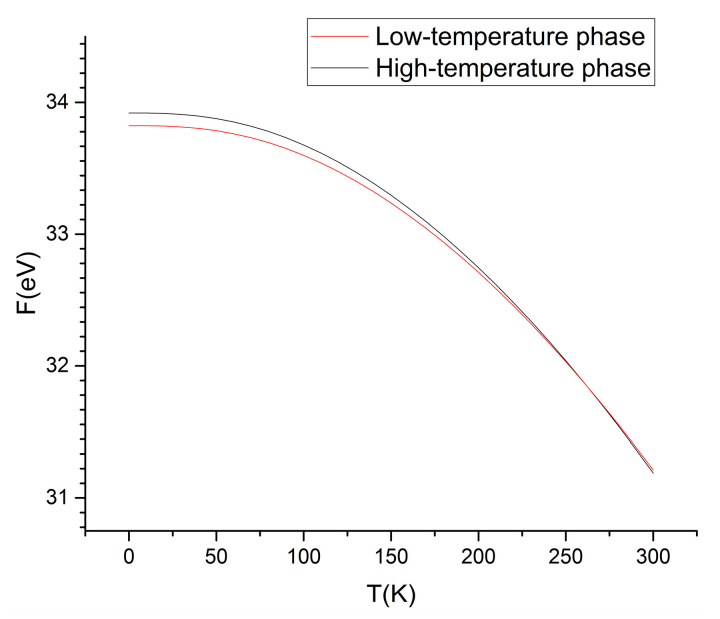
Calculated dependence of Helmholtz free energy on temperature. Curves intersect at 263 K, which is near the phase transition temperature reported in the literature (258.15 K).

Another way to find ADPs is to calculate them from the phonon spectra. This way can only calculate relative ADPs, but the main axes of the most intensive harmonic oscillations should have the same direction as in the experiment. Phonon spectra for low- and high-temperature cells almost match, but several peaks are shifted. These signals are located at ranges from 400 to 550 cm^−1^ and from 950 to 1100 cm^−1^ and respond to rocking oscillations of the siloxane ring (Figure 6). Oscillations with wavenumbers between 400 and 550 cm^−1^ also move molecular centroids in the same direction as an intensive oscillation in the MD calculations. The multipeak at 1050 cm^−1^ corresponds to the rotational oscillation of the siloxane ring. Many thermodynamic properties (S, C_v_, F, etc.) of the crystal phases can be calculated from phonon spectra. The dependence of Helmholtz free energy on temperature is shown in Figure 7. According to the calculation, at temperature 258 K, ΔF is equal to 0.08 kcal/mol.

### 2.5. Phase Transition Simulation

**Figure 8:**
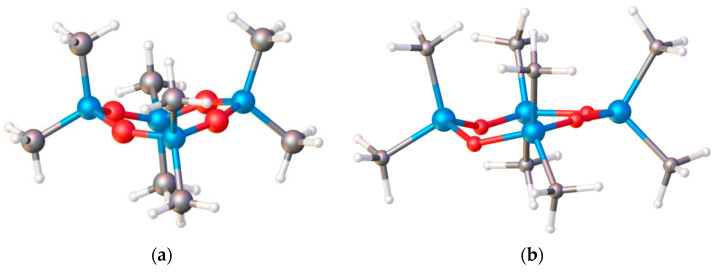
Transition state conformation of an isolated D4 molecule (**a**) and a conformation found in the local minimum by MD calculation (**b**). Color code: silicon—blue, oxygen—red, carbon—grey, hydrogen—white.

Phase transition analysis started with the calculation of transition state conformation. First, we sought to determine the transition state by using the Berny algorithm [24] and an intrinsic reaction coordinate [20] calculation in the Gaussian 09 program. Unfortunately, the potential energy surface (PES) of a set of conformations is very flat, and only approximate conformation of transition state can be calculated. The calculated conformation was boat–boat (D_2d_ symmetry, Figure 8a). The Si1-Si2-Si3-Si4 torsion angle equals 145.35°. Si-O-Si angles are 151.44, 153.84, 153.90, and 158.58°. The tetrahedral environment of silicon atoms persists (O-Si-O angle = 109.45(5)°). Each pair of oxygen atoms was very close to the plane formed by the three nearest silicon atoms (distances to the plane are less than 0.05 Å). The calculated difference between low-temperature conformation energy and calculated transition state energy was 0.07 kcal/mol. This very small value is the activation barrier of the conformation transition of an isolated molecule.

The phase transition energy barrier in a real crystal could be much higher and can only be calculated correctly using a crystal environment and periodic boundary conditions. Thus, the next step to obtain a phase transition pathway was restrained molecular dynamics with periodical boundary conditions. This calculation was carried out with VASP software. Several dihedral angles and nonbonding distances were restrained during molecular dynamics calculation. This time, the conformational pathway found had an unbelievably high energy barrier. This result was obtained due to the abundance of restraints, leading to the collapse of the molecule. The gradual weakening of imposed restrictions solved this problem. When only the internal coordinates of the molecules were restricted, the best result (most equilibrium pathway) was achieved.

Finally, the best but the most time-consuming way is to the trace the transition path from the molecular dynamics’ simulation. The character of molecular movement in a crystal was found and it was reproduced in several separate MD simulations. The oscillation had a period of 4 ps and existed between 210 K and 270 K (inclusive). Several molecules (one or two of four, presented in a unit cell) in a crystal move up to 1.6 Å along c axes, which correlates well with the distance of average shift performed by molecules during phase transition. We can assume that two types of molecular translational movement within a unit cell are possible. The first one implies correlated movement of molecules. The second type is the situation when molecules move intendedly. In this case, we observed that one of four molecules move translationally when others conserve their position despite temperature changes from 210 to 270 K. Such character of molecular movement can be considered asynchronous.

The MD calculations concluded with a notable result: a certain region in the PES was limited by energy borders. All molecules in this “crystal” state had taken different conformations even in a 2 × 2 × 1 supercell. Nevertheless, the conformations of D4 molecules remain similar: at least a half cycle of the molecule is almost flat (Si-O-Si-O-Si chain, Figure 8b). Molecules can change their conformations without leaving a minimum in the PES, and since the ring could become almost flat, they will be able to take the conformation of both high- and low-temperature phases. Thus, the wide flat local minimum of energy cannot be assigned to any of the known crystal phases and could be considered as an amorphous phase.

The addition of bias potential to the molecular dynamics calculations makes it possible to force the system to undergo a phase transition. This allows us to trace one of the transition paths, but, obviously, not the most optimal one. The energy parameters of the phase transition also cannot be determined from the result of molecular dynamics using the bias potential. Nevertheless, the use of the bias potential made it possible to check how well the chosen coordinate corresponds to the process—in this case, the phase transition.

**Figure 9:**
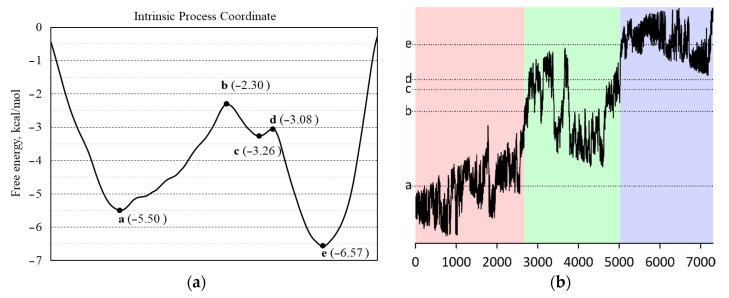
(**a**) The dependence of free energy on intrinsic coordinate during the phase transition in crystal of D4. The high-temperature phase is on the left, and the low-temperature phase is on the right side of plot. (**b**) The path of intrinsic coordinate (vertical) during metadynamics.

Born–Oppenheimer molecular dynamics calculations (with and without bias potential) have demonstrated the possibility of simulating a phase transition in a crystal by computational methods. However, the phase transition does not have to go through a metastable phase. To establish the energy parameters of the phase transition, as well as to verify the assumption that the metastable phase is a local minimum in the PES, we applied a recent computational approach that has rarely been used to study processes of this type: ab initio metadynamics. Metadynamics made it possible to recognize the dependence of the free energy on the intrinsic coordinate of the process. The intrinsic coordinate of the process is a linear combination of the distances between atoms in the system and was chosen so that the values on the left refer to the system in the high-temperature phase (Figure 9a, point a), and the values on the right relate to the low-temperature phase (Figure 9a, point e). Point c in the diagram (Figure 9a) relates to metastable phase. The calculated energy barrier was only 3.20 kcal/mol during the transition from the high-temperature phase to the low-temperature phase, and 4.27 kcal/mol in the opposite direction at an average temperature of 238 K. The disordered phase found in Born–Oppenheimer molecular dynamics calculations also occurs in metadynamics. In the figure, it is represented by the roughness of the right slope of the high-temperature phase (segment a–b in Figure 9a). The free energy change during such a transition should be about 1.07 kcal/mol. Considering the DSC data (1.04(7) kcal/mol), the entropy of studied phases should be almost equal.

The path of intrinsic coordinate during metadynamics can be divided into several areas (Figure 9b). The first area (red, points 0–2700) corresponds to a high-temperature phase and metastable states. The stepwise character of the coordinate change is caused by the existence of several states that are stable under these conditions. In the next area (green, 2700–4600), the crystal system oscillates near point b, passing to the minimum c and returning to the metastable state a–b. After point 5050, the system crosses points b, c, and d and moves into the low-temperature phase. The calculation was terminated when conformations of molecules became unrealistic (the bond lengths (+/− 0.07 Å) and bond angles (+/−30 °) were distorted).

To prove that metadynamics had completed, additional calculations were made using the obtained bias potentials. The calculations were molecular dynamics with tracking of the intrinsic coordinate. The starting point of the calculation was the state of the system at every 1000th step. The system behaved chaotically, in some cases tending to leave the area of interest, bending the molecules into unrealistic conformations. Rarely, the system is stuck near point c, which may indicate an error in the calculated energy. However, since the nature of being stuck was random and rarely encountered, it was decided that the metadynamics could be considered complete.

## 3. Materials and Methods

### 3.1. X-ray Experimental Studies

**Figure 10:**
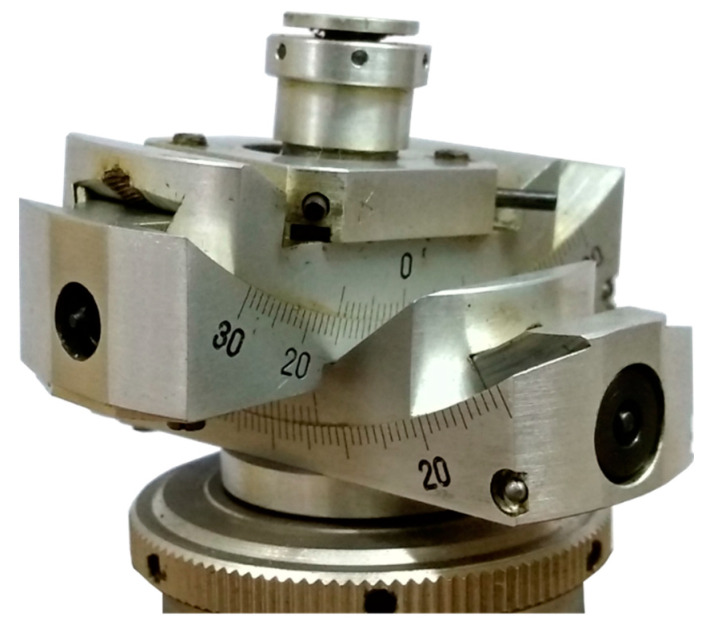
A special goniometer head was designed and manufactured for in situ X-ray studies.

Single crystals of D4 were grown in a sealed glass capillary (0.3 mm diameter) upon slow crystallization using nichrome wire as a heater. Crystallization was performed at 270 K. The movement of the heater along the glass capillary was maintained by a hand-made elevating unit with PC control. We performed 3 experimental series. Cooling rates between X-ray experiments were 60, 30, 20, and 15 °C/h. All equipment for crystal growth was installed into a Bruker APEX II diffractometer with on three-circle Eulean goniometer with a fixed χ axis (Bruker AXS, Karlsruhe, Germany). Cobra (Oxford Cryosystems, Long Hanborough, England) was used for temperature control. To simplify crystal growth on a three-circle goniometer, a special goniometer head was designed and manufactured (Figure 10). With this head, it became possible to turn the capillary into a vertical position from an inclined one (~54.7°) and vice versa. In fact, all crystals grown by this technique consisted of a main domain with several small ones. However, it was possible to distinguish the diffraction patterns for various domains using RLATT and CELL_NOW software (Bruker AXS, Karlsruhe, Germany) [30].

To solve and refine the structures of low- and high-temperature phases of D4 measured at various temperatures, the SHELX-2014 program package (by Sheldrick, Göttingen, Germany [31]) was utilized. All the structures were solved by the intrinsic phasing method (SHELXT program, version 2014/4 [31]) and refined with the least-squares method (SHELXL program, version 2014/6 [31]) in anisotropic approximation for non-hydrogen atoms. The positions of hydrogen atoms were refined using a riding model (AFIX 137 instruction). Crystallographic data and information about the refinement of crystal structures can be found in Supplementary Materials (Table S1)). The Cambridge Crystallographic Data Centre (CCDC 2,182,853–2,182,861) contains the supplementary crystallographic data for the D4 crystal at 100, 150, 200, 210, 221, 230, 240, 250, and 270 K. These data can be obtained free of charge from The Cambridge Crystallographic Data Centre via https://www.ccdc.cam.ac.uk/structures (accessed on 2 August 2022).

### 3.2. Differential Scanning Calorimetry

Differential scanning calorimetry (DSC) analyses were provided on DSC25 (TA Instruments, New Castle, DE, USA) with the cooling system RCS 90. TRIOS Software (TA Instruments, New Castle, USA) was used as a hardware control program. The weighed portions were encapsulated in aluminum pot Tzero (CS Ceramic Co., Ltd., Chashan Town, Liling City, Hunan Province, China). The range of scanned temperatures was from −80 °C to 60 °C. Temperature change rates were 2, 3, 5, and 7 °C/min.

### 3.3. Calculations

Ab initio calculations of molecular dynamics and metadynamics were performed within the PBE exchange-correlation functional using Vienna Ab initio Simulation Package v. 5.4.1 (VASP, VASP Software GmbH, Vienna, Austria) [32,33]. A combination of pseudopotentials and a set of plane waves was used as a basis set. To improve the description of van der Waals interactions, the D3 correction was applied. The point group symmetry within a unit cell was switched off during all calculations, including molecular dynamics simulations. The atomic cores were described using the “ultrasoft” and “hard” projector augmented wave (PAW) potentials. The basis of plane waves was used to describe valence electrons: the kinetic energy limit was 300 (for ultrasoft pseudopotentials) or 800 eV (for “hard” pseudopotentials). To describe the temperature of the system, calculations were carried out using a Nose–Hoover thermostat [34,35]. The starting point of the calculation corresponds to the experimentally obtained data (elementary cell parameters, atomic positions, and temperature). The time integration step was chosen to be 1 fs (femtosecond).

Born–Oppenheimer molecular dynamics calculations were carried out using “hard” pseudopotentials and a kinetic energy limit of 800 eV. The same applies to molecular dynamics calculations using internal constraints. To study the path of the phase transition, the complex coordinate of the process (weighted sum of interatomic distances) was changed by a small value with each time step. The cell parameters were fixed at the maximum experimental values reached during the phase transition.

The parameters of the anisotropic displacement of atoms were calculated from the trajectories of atoms obtained in molecular dynamics calculations without restrictions. Trajectories of the motion of atoms from 1 to 10,000 steps (the first 10 ps) were not taken into account in this calculation. The analysis of a large amount of data on the movement of atoms was carried out by an application program written personally by the author to solve this problem.

To carry out metadynamics calculations, ultrasoft pseudopotentials and a kinetic energy limit of 300 eV were used. The weighted sum of intra- and intermolecular distances between atoms was used as the process coordinate. The calculation was carried out until the system began to randomly change its state. The total duration of the calculation was 800,000 steps or 800 ps. The dependence of the free energy on the process coordinate was calculated based on 7300 Gaussian functions with a height of 0.005 eV and a width of 0.2 Å.

The molecular dynamics (MD) calculations were performed using the VASP for D4 crystal lattices at 100, 150, 200, 210, 221, 230, 240, 250, and 270 K. The MD simulations started from the corresponding X-ray structures. The periodic boundary conditions with the NVT ensemble (Nose–Hoover thermostat) were used. Trajectories of 15 ps were collected after a 5 ps equilibration period, with 1 fs integration time.

The transition state conformation was determined using the Berny algorithm [24] and an intrinsic reaction coordinate [20] calculation in Gaussian 09 program (rev. A.01, Gaussian, Inc., Wallingford, CT, USA). The exchange-correlation PBE0 functional with the basis 6–311 G(d,p) was used for calculations [36].

Phonon spectrum calculations for 100 K and 250 K cell units were provided with the PHON program (version 1.43, by Dario Alfè, London, UK) [37]. From the phonon frequencies, we can calculate the dependence of the Helmholtz free energy on temperature and atom displacement parameters.

### 3.4. Mathematical Introduction and Theory

Atomic displacement parameters (ADPs) for each atom can be calculated from MD trajectories as elements of the covariance matrix:

(1) $$U_{ij}=\;\langle (X_{i}-\langle X_{i}\rangle )\;\left(X_{j}-\langle X_{j}\rangle \right)\rangle \;,$$

where *X_i_* and *X_j_* are the *x*, *y*, or *z* coordinates in a Cartesian coordinate system and angular brackets denote averaging over time, i.e., over all points of the trajectory.

The value of the equivalent displacement in orthogonal coordinate systems equals one-third of a trace of the atomic displacement matrix $U_{ij}$ and represents an average mean square displacement of the atom over three main directions:

(2) $$U_{eq}=\frac{1}{3}\left(U_{11}+U_{22}+U_{33}\right),$$

where $U_{11}$, $U_{22}$, and $U_{33}$ are the mean square displacements in the *x*, *y,* and *z* directions, respectively.

The average ADPs by atom type were calculated according to (2) and then compared to experimental ones:

(3) $$\langle U_{eq}\left(t,T\right)\rangle =\frac{\sum_{i}U_{eq}\left(a_{i},T\right)}{N_{t}},$$

where *a_i_* is the type of atom (carbon, oxygen, etc.), *T* is the temperature, and $N_{t}$ is the number of atoms of type *t*. Angular brackets denote the average value over all atoms of the same type.

## 4. Conclusions

The temperature-dependent series of X-ray diffraction experiments with octamethylcyclotetrasiloxane (D4) made it possible to reliably establish the dependence of the cell parameters and atomic positions on temperature. Both indicated phases were determined at several temperatures. The large hysteresis loop on DSC and the slow-cooling crystal destruction phenomenon may be a consequence of the existence of a metastable phase. The Born–Oppenheimer molecular dynamics simulation of phase transition shows the possibility of the presence of an intermediate amorphous phase. Calculating ADPs from atom trajectories could easily detect a presence of anomalous behavior in MD calculations. During all unrestricted molecular dynamics calculations, the system in the high-temperature phase changed into an unknown unsymmetrical phase. The simulated metadynamics of phase transition shows a presence of two different metastable phases. Thus, the phase transition is asynchronous and could pass through unstable amorphous phases. The comparison of easily obtained phonon spectra can help to find changes in the molecular environment and to describe the first steps of a phase transition pathway.

## Figures and Tables

**Table 1:** Average equivalent ADPs by temperature and type of atom.

Temperature, K	$\mathrm{Experimental}\;\langle U_{eq}\left(t,T\right)\rangle \times 10^{3}$			$\mathrm{Calculated}\;\langle U_{eq}\left(t,T\right)\rangle \times 10^{3}$		
	Si	O	C	Si	O	C
100	16.515	21.937	26.866	17.838	28.643	34.753
150	23.682	30.892	38.581	22.447	32.650	40.655
200	30.027	39.202	49.814	30.779	42.338	57.780
210	31.883	41.93	53.364	37.851	47.740	89.316
221	34.277	44.518	56.858	38.193	53.145	74.892
230	35.827	48.093	60.622	75.543	93.211	142.686
240	54.897	69.820	109.33	65.665	97.464	134.831
250	57.437	72.773	114.994	56.689	68.092	151.815
270	68.383	85.827	131.237	45.922	70.727	107.603

## Data Availability

The data presented in this study are available on request from the corresponding author.

## Supplementary Materials

The supporting information can be downloaded at https://www.mdpi.com/article/10.3390/ijms23169073/s1.
