# Supplementary material for: Various Approaches to Studying the Phase Transition in an Octamethylcyclotetrasiloxane Crystal: From X-ray Structural Analysis to Metadynamics
Source: Int J Mol Sci. 2022 Aug 13;23(16):9073. doi: 10.3390/ijms23169073 (PMC9408834; doi:10.3390/ijms23169073)

checkCIF/PLATON report

Structure factors have been supplied for datablock(s) 210

THIS REPORT IS FOR GUIDANCE ONLY. IF USED AS PART OF A REVIEW PROCEDURE FOR PUBLICATION, IT SHOULD NOT REPLACE THE EXPERTISE OF AN EXPERIENCED CRYSTALLOGRAPHIC REFEREE.

No syntax errors found.   CIF dictionary   Interpreting this report

Datablock: 210

```
Bond precision:   Si- C = 0.0038 A         Wavelength=0.71073

Cell:               a=16.051(2)      b=16.051(2)      c=6.4589(18)
                    alpha=90         beta=90          gamma=90
Temperature:        210 K

                 Calculated                     Reported
Volume           1664.0(6)                      1664.0(6)
Space group      P 42/n                         P 42/n
Hall group       -P 4bc                         -P 4bc
Moiety formula   C8 H24 O4 Si4                  C8 H24 O4 Si4
Sum formula      C8 H24 O4 Si4                  C8 H24 O4 Si4
Mr               296.63                         296.63
Dx,g cm-3        1.184                          1.184
Z                4                              4
Mu (mm-1)        0.355                          0.355
F000             640.0                          640.0
F000'            641.51
h,k,lmax         23,23,9                        23,20,9
Nref             2581                           2557
Tmin,Tmax        0.880,0.899                    0.558,0.746
Tmin'            0.837

Correction method= # Reported T Limits: Tmin=0.558 Tmax=0.746
AbsCorr = MULTI-SCAN

Data completeness= 0.991            Theta(max)= 30.644

                                                        wR2(reflections)=
R(reflections)= 0.0611( 1372)                           0.1596( 2557)

S = 1.042                   Npar= 77
```

The following ALERTS were generated. Each ALERT has the format
test-name_ALERT_alert-type_alert-level.
Click on the hyperlinks for more details of the test.

Alert level C

```
PLAT242_ALERT_2_C Low    'MainMol' Ueq as Compared to Neighbors of        Si2 Check
PLAT906_ALERT_3_C Large K Value in the Analysis of Variance ......     14.081 Check
PLAT906_ALERT_3_C Large K Value in the Analysis of Variance ......      2.587 Check
PLAT911_ALERT_3_C Missing FCF Refl Between Thmin & STh/L=    0.600         18 Report
```

Alert level G

```
PLAT019_ALERT_1_G _diffrn_measured_fraction_theta_full/*_max < 1.0      0.997 Report
PLAT764_ALERT_4_G Overcomplete CIF Bond List Detected (Rep/Expd) .       1.12 Ratio
PLAT912_ALERT_4_G Missing # of FCF Reflections Above STh/L=  0.600          6 Note
PLAT933_ALERT_2_G Number of OMIT Records in Embedded .res File ...          3 Note
PLAT941_ALERT_3_G Average HKL Measurement Multiplicity ..........         4.0 Low
PLAT951_ALERT_5_G Calculated (ThMax) and CIF-Reported Kmax Differ           3 Units
```

0 **ALERT level A** = Most likely a serious problem - resolve or explain
0 **ALERT level B** = A potentially serious problem, consider carefully
4 **ALERT level C** = Check. Ensure it is not caused by an omission or oversight
6 **ALERT level G** = General information/check it is not something unexpected

1 ALERT type 1 CIF construction/syntax error, inconsistent or missing data
2 ALERT type 2 Indicator that the structure model may be wrong or deficient
4 ALERT type 3 Indicator that the structure quality may be low
2 ALERT type 4 Improvement, methodology, query or suggestion
1 ALERT type 5 Informative message, check

It is advisable to attempt to resolve as many as possible of the alerts in all categories. Often the minor alerts point to easily fixed oversights, errors and omissions in your CIF or refinement strategy, so attention to these fine details can be worthwhile. In order to resolve some of the more serious problems it may be necessary to carry out additional measurements or structure refinements. However, the purpose of your study may justify the reported deviations and the more serious of these should normally be commented upon in the discussion or experimental section of a paper or in the "special_details" fields of the CIF. checkCIF was carefully designed to identify outliers and unusual parameters, but every test has its limitations and alerts that are not important in a particular case may appear. Conversely, the absence of alerts does not guarantee there are no aspects of the results needing attention. It is up to the individual to critically assess their own results and, if necessary, seek expert advice.

Publication of your CIF in IUCr journals

A basic structural check has been run on your CIF. These basic checks will be run on all CIFs submitted for publication in IUCr journals (*Acta Crystallographica*, *Journal of Applied Crystallography*, *Journal of Synchrotron Radiation*); however, if you intend to submit to *Acta Crystallographica Section C* or *E* or *IUCrData*, you should make sure that full publication checks are run on the final version of your CIF prior to submission.

Publication of your CIF in other journals

Please refer to the *Notes for Authors* of the relevant journal for any special instructions relating to CIF submission.

PLATON version of 13/07/2021; check.def file version of 13/07/2021

Datablock 210 - ellipsoid plot

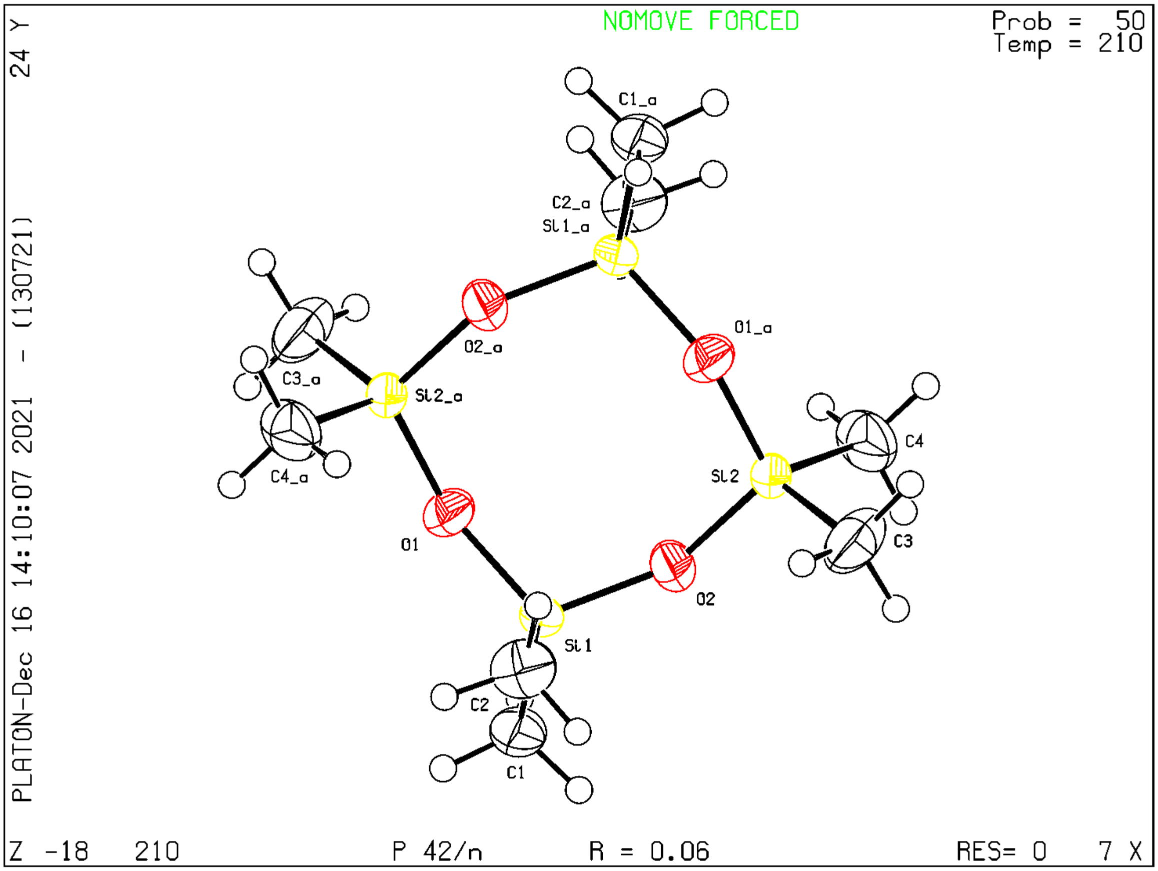

Supplement: Supplementary file 1 [file ijms-23-09073-s001.zip › 1-11_210_cifreport.pdf]
