# Supplementary material for: Various Approaches to Studying the Phase Transition in an Octamethylcyclotetrasiloxane Crystal: From X-ray Structural Analysis to Metadynamics
Source: Int J Mol Sci. 2022 Aug 13;23(16):9073. doi: 10.3390/ijms23169073 (PMC9408834; doi:10.3390/ijms23169073)

checkCIF/PLATON report

Structure factors have been supplied for datablock(s) 240

THIS REPORT IS FOR GUIDANCE ONLY. IF USED AS PART OF A REVIEW PROCEDURE FOR PUBLICATION, IT SHOULD NOT REPLACE THE EXPERTISE OF AN EXPERIENCED CRYSTALLOGRAPHIC REFEREE.

No syntax errors found. CIF dictionary Interpreting this report

Datablock: 240

```
Bond precision:   Si- C = 0.0040 A        Wavelength=0.71073

Cell:              a=16.0850(16)      b=16.0850(16)      c=6.8911(9)
                   alpha=90           beta=90            gamma=90
Temperature:       240 K

                Calculated                       Reported
Volume          1782.9(4)                        1782.9(4)
Space group     I 41/a                           I 41/a
Hall group      -I 4ad                           -I 4ad
Moiety formula  C8 H24 O4 Si4                    C8 H24 O4 Si4
Sum formula     C8 H24 O4 Si4                    C8 H24 O4 Si4
Mr              296.63                           296.63
Dx,g cm-3       1.105                            1.105
Z               4                                4
Mu (mm-1)       0.331                            0.331
F000            640.0                            640.0
F000'           641.51
h,k,lmax        23,23,9                          23,19,9
Nref            1383                             1376
Tmin,Tmax       0.888,0.905                      0.560,0.746
Tmin'           0.847

Correction method= # Reported T Limits: Tmin=0.560 Tmax=0.746
AbsCorr = MULTI-SCAN

Data completeness= 0.995           Theta(max)= 30.714

R(reflections)= 0.0545( 816)                           wR2(reflections)=
                                                       0.1724( 1376)
S = 1.090                    Npar= 39
```

The following ALERTS were generated. Each ALERT has the format
test-name_ALERT_alert-type_alert-level.
Click on the hyperlinks for more details of the test.

Alert level C

PLAT242_ALERT_2_C Low 'MainMol' Ueq as Compared to Neighbors of Si1 Check

Alert level G

PLAT764_ALERT_4_G Overcomplete CIF Bond List Detected (Rep/Expd) . 1.25 Ratio
PLAT912_ALERT_4_G Missing # of FCF Reflections Above STh/L= 0.600 7 Note
PLAT941_ALERT_3_G Average HKL Measurement Multiplicity .... 4.6 Low
PLAT951_ALERT_5_G Calculated (ThMax) and CIF-Reported Kmax Differ 4 Units

0 **ALERT level A** = Most likely a serious problem - resolve or explain
0 **ALERT level B** = A potentially serious problem, consider carefully
1 **ALERT level C** = Check. Ensure it is not caused by an omission or oversight
4 **ALERT level G** = General information/check it is not something unexpected

0 ALERT type 1 CIF construction/syntax error, inconsistent or missing data
1 ALERT type 2 Indicator that the structure model may be wrong or deficient
1 ALERT type 3 Indicator that the structure quality may be low
2 ALERT type 4 Improvement, methodology, query or suggestion
1 ALERT type 5 Informative message, check

It is advisable to attempt to resolve as many as possible of the alerts in all categories. Often the minor alerts point to easily fixed oversights, errors and omissions in your CIF or refinement strategy, so attention to these fine details can be worthwhile. In order to resolve some of the more serious problems it may be necessary to carry out additional measurements or structure refinements. However, the purpose of your study may justify the reported deviations and the more serious of these should normally be commented upon in the discussion or experimental section of a paper or in the "special_details" fields of the CIF. checkCIF was carefully designed to identify outliers and unusual parameters, but every test has its limitations and alerts that are not important in a particular case may appear. Conversely, the absence of alerts does not guarantee there are no aspects of the results needing attention. It is up to the individual to critically assess their own results and, if necessary, seek expert advice.

Publication of your CIF in IUCr journals

A basic structural check has been run on your CIF. These basic checks will be run on all CIFs submitted for publication in IUCr journals (*Acta Crystallographica*, *Journal of Applied Crystallography*, *Journal of Synchrotron Radiation*); however, if you intend to submit to *Acta Crystallographica Section C* or *E* or *IUCrData*, you should make sure that full publication checks are run on the final version of your CIF prior to submission.

Publication of your CIF in other journals

Please refer to the *Notes for Authors* of the relevant journal for any special instructions relating to CIF submission.

PLATON version of 13/07/2021; check.def file version of 13/07/2021

Datablock 240 - ellipsoid plot

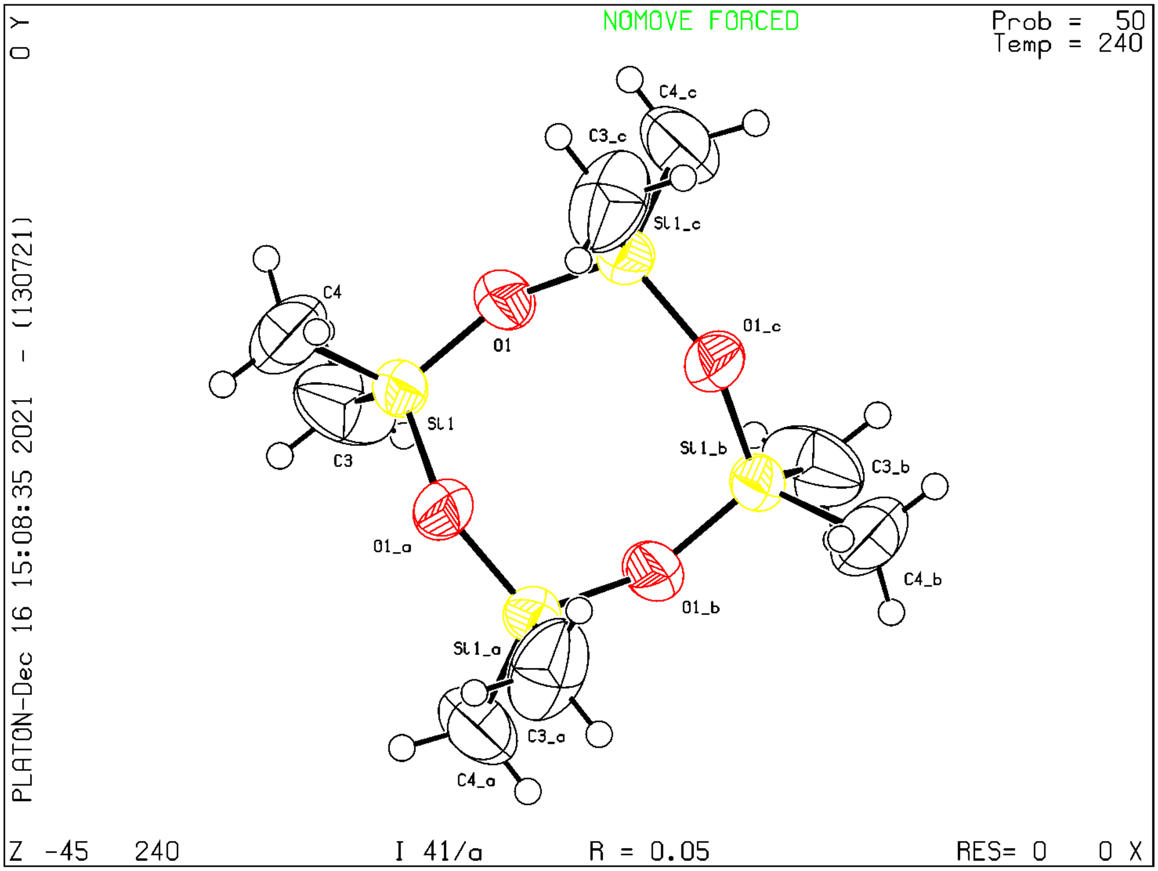

Supplement: Supplementary file 1 [file ijms-23-09073-s001.zip › 1-11_240_cifreport.pdf]
