# Supplementary material for: Various Approaches to Studying the Phase Transition in an Octamethylcyclotetrasiloxane Crystal: From X-ray Structural Analysis to Metadynamics
Source: Int J Mol Sci. 2022 Aug 13;23(16):9073. doi: 10.3390/ijms23169073 (PMC9408834; doi:10.3390/ijms23169073)
Supplement: Supplementary file 1 [file ijms-23-09073-s001.zip › ESI.pdf]

Electronic supporting information (ESI) for article: Various Approaches to Studying the Phase Transition in an Octamethylcyclotetrasiloxane Crystal: from X-Ray Structural Analysis to Metadynamics

Alexander D. Volodin[1,2,*], Alexander F. Smol'yakov[1,3], Alexander A. Korlyukov[1,2]

[1] *A. N. Nesmeyanov Institute of Organoelement Compounds, Russian Academy of Science, Vavilova str. 28, 119991, Moscow, Russian Federation;*

[2] *D. Mendeleev University of Chemical Technology of Russia, Miusskaya sq. 9, 125047, Moscow, Russian Federation;*

[3] *Plekhanov Russian University of Economics, Stremyanny per. 36, 117997, Moscow, Russian Federation;*

* Correspondence: alex.d.volodin@gmail.com;

Keywords: *in situ* cryo-crystallization; X-ray structure determination; periodic DFT calculations; quantum chemical calculations; Born-Oppenheimer molecular dynamics; metadynamics; phase transition path; free energy of phase transition.

Table S1. Crystallographic data of octamethylcyclotetrasiloxane at different temperatures.

Temperature/K	100	150	200	210	221	230	240	250	270
Crystal system	tetragonal	tetragonal	tetragonal	tetragonal	tetragonal	tetragonal	tetragonal	tetragonal	tetragonal
Space group	$P4_2/n$	$P4_2/n$	$P4_2/n$	$P4_2/n$	$P4_2/n$	$P4_2/n$	$I4_1/a$	$I4_1/a$	$I4_1/a$
a/Å	15.9596(16)	15.9938(15)	16.0373(19)	16.051(2)	16.0690(19)	16.083(3)	16.0850(16)	16.0850(16)	16.250(7)
b/Å	15.9596(16)	15.9938(15)	16.0373(19)	16.051(2)	16.0690(19)	16.083(3)	16.0850(16)	16.0850(16)	16.250(7)
c/Å	6.3737(15)	6.4099(14)	6.4481(17)	6.4589(18)	6.4719(17)	6.517(3)	6.8911(9)	6.8911(9)	6.943(4)
α/°	90	90	90	90	90	90	90	90	90
β/°	90	90	90	90	90	90	90	90	90
γ/°	90	90	90	90	90	90	90	90	90
Volume/Å^3	1623.4(5)	1639.7(5)	1658.4(6)	1664.0(6)	1671.1(6)	1685.7(10)	1782.9(4)	1782.9(4)	1833.3(19)
Z	4	4	4	4	4	4	4	4	4
ρ_{calc}g/cm^3	1.214	1.202	1.188	1.184	1.179	1.169	1.105	1.105	1.075
μ/mm^{-1}	0.364	0.360	0.356	0.355	0.354	0.350	0.331	0.331	0.322
2Θ range for data collection/°	3.61 to 61.564	3.602 to 61.25	3.592 to 61.196	3.588 to 61.288	3.584 to 61.316	3.582 to 61.18	5.064 to 61.428	5.064 to 61.536	5.014 to 51.988
Reflections collected	5755	10212	10231	10350	10524	9559	6271	6282	2110
R_{int}	0.0313	0.0444	0.0522	0.0548	0.0520	0.0734	0.0398	0.0382	0.0471
Data/restraints/parameters	2160/0/77	2511/0/77	2530/0/77	2557/0/77	2572/0/77	2557/0/77	1376/0/39	1381/0/39	886/0/39
Goodness-of-fit on F^2	1.072	1.034	1.032	1.042	1.001	1.066	1.090	1.087	1.043
R_1 [I>=2σ (I)]	0.0477	0.0472	0.0572	0.0611	0.0598	0.0883	0.0545	0.0560	0.0907
wR_2 [all data]	0.1220	0.1295	0.1553	0.1596	0.1599	0.2423	0.1724	0.1906	0.2999
Largest diff. peak/hole / e Å^{-3}	0.43/-0.26	0.36/-0.24	0.44/-0.22	0.42/-0.26	0.42/-0.25	0.51/-0.34	0.39/-0.28	0.45/-0.30	0.72/-0.41

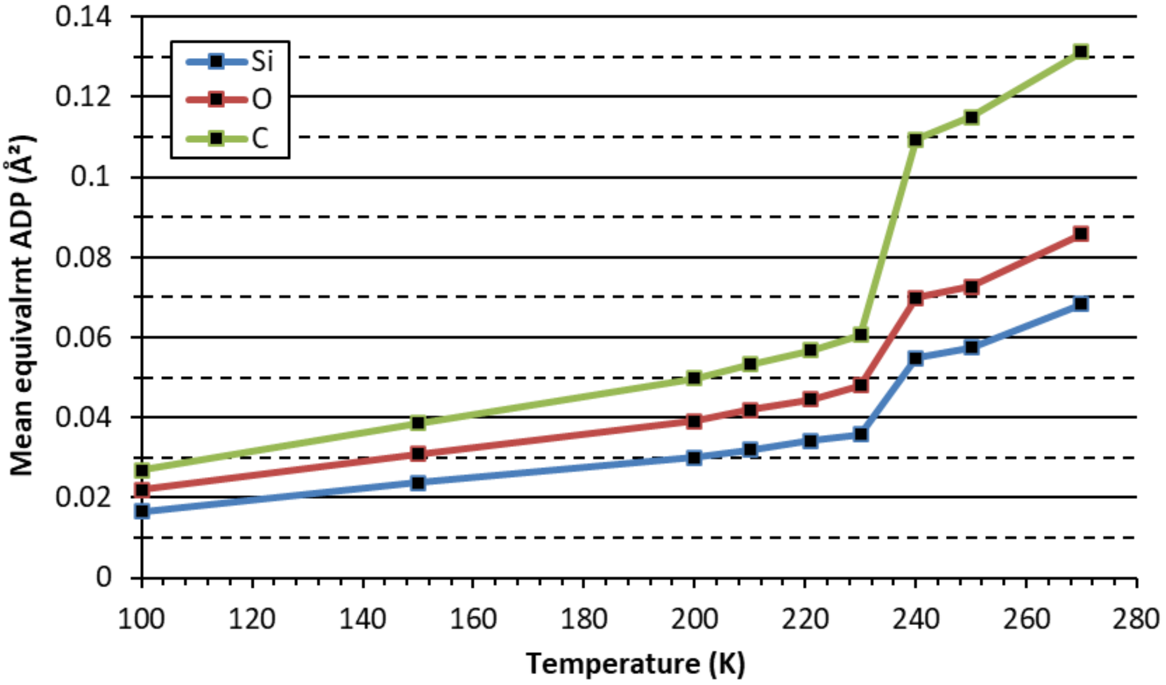

Figure S1. Isotropic atom displacement parameters (U_{eq}) averaged by atom type.
